# Controlling Effects of Nanocomposite Sterilant ND-1 on the Growth of Wild Populations of Midday Gerbil (*Meriones meridianus*)

**DOI:** 10.3390/life13122280

**Published:** 2023-11-29

**Authors:** Fan Bu, Xin Li, Junyuan Bai, Shanshan Sun, Haiwen Yan, Haoting Zhang, Yongling Jin, Linlin Li, Rong Zhang, Zhenghaoni Shang, Shuai Yuan, Xiaodong Wu, Heping Fu

**Affiliations:** 1 College of Grassland Resources and Environment, Inner Mongolia Agricultural University, Hohhot 010011, China; bufan@emails.imau.edu.cn (F.B.); lixin9623@163.com (X.L.); sunshanshan@emails.imau.edu.cn (S.S.); yanhaiwen@emails.imau.edu.cn (H.Y.); zhanghaoting@emails.imau.edu.cn (H.Z.); jinyongling@nmgnydx61.wecom.work (Y.J.); lilinlin9630@emails.imau.edu.cn (L.L.); zhangrong20212021@126.com (R.Z.); shangzhn1997@emails.imau.edu.cn (Z.S.); wuxiaodong_hgb@163.com (X.W.); 2 Key Laboratory of Grassland Rodent Ecology and Pest Controlled, Hohhot 010011, China; 3 Key Laboratory of Grassland Resources, Ministry of Education, Hohhot 010011, China; 4 Horqin Right Front Banner Science and Technology Development Center, Xing’an League, Ulanhot 137400, China; 13141186066@163.com

**Keywords:** compound sterility, nanometre, midday gerbil, wild populations, desert area

## Abstract

Grassland is not only an important part of the terrestrial ecosystem with multiple ecological functions, but also an important base for Chinese herdsmen to produce and live. However, the occurrence and spread of rodent infestation reduces the biodiversity and productivity of grassland ecosystems. It also severely threatens human life, health, and biosecurity through disease transmission. In this study, we explored the ability of the nanocomposite sterilant ND-1 to control grassland rodent populations. Semi-closed experimental and control plots were established in the desert area of Alashan, Inner Mongolia, China. In spring 2018, the nanocomposite sterile ND-1 (Nongda-1) was introduced once, and the control effect of ND-1 on the growth of the wild population of midday gerbils was measured for two years. We show that ND-1 significantly reduced the population of midday gerbils in the experimental area, with a negative population growth rate. In addition, in the second year, the ratio of female midday gerbils to sub-adults in the experimental area was significantly lower than that in the control area, which resulted in a significant difference in the sex ratio of midday gerbils. There were significantly fewer females than males, and the population growth of midday gerbils in the experimental area was significantly inhibited. ND-1 had no significant effect on the home range of midday gerbils, and sterile individuals continued to occupy the home range and consume resources. Therefore, ND-1 reduced the number of female midday gerbils during the breeding period and the sex ratio and population density and altered the age structure of the wild population. Additionally, competition between sterile and normal individuals had a significant control effect on the growth of wild populations. Our studies demonstrate the significance of ND-1 in the sustainable control of grassland rodent pests, with the potential for limiting grassland rodent damage in the future.

## 1. Introduction

Rodent pests have become a prominent problem facing the world today [1,2,3,4,5]. Crop losses caused by rodents account for approximately 10–20% of the total output value annually worldwide [6]. Some rodents are hosts and vectors of natural focal diseases that can carry a variety of pathogens and spread zoonoses, such as plague, leptospirosis, and epidemic hemorrhagic fever [7,8]. The emergence of grassland rodent infestations in particular has seriously threatened public health and safety, biosecurity, grassland ecological environment construction, and the sustainable development of grassland animal husbandry [9,10]. In recent years, chemical bait killing has been the primary method used to control grassland rodents. This has the advantages of having a rapid effect and a low cost and plays an important role in grassland rodent control technology [11]. However, chemical drugs easily pollute the environment and are less selective for pest rodents. This can cause secondary poisoning and bioconcentration phenomena, and accidentally injure many non-target animals [12]. This then leads to a decline in natural enemy rodent species and population sizes. This, in turn, leads to a reduction in biodiversity and the structure and function of ecosystems. After long-term use, rodents develop drug resistance that does not fundamentally change their habits and produces an over-compensation effect. Rodent populations will quickly return to their original levels [13,14,15,16]. Fertility control has the potential to solve this problem [17]. The use of plant-derived extracts to control fertility in rodents can help achieve sustainable goals and help environments to become pollution-free.

At present, some chemical and hormonal sterile agents have shown pronounced effects in rodent tests [18,19]. However, some problems remain, such as the palatability of bait types to rodents in the wild and the degree of environmental degradation [19]. There have also been some reports on natural plants and their extracts that have had fertility effects on mammals, such as *Curcuma zedoaria* and *Arnebia euchroma*, which have had fertility effects on female laboratory mice (*Mus musculus*) [20,21]. *Tripterygium wilfordii* and *Sophora flavescens* have had sterile effects on male laboratory mice [22]. An ideal sterilant should be sterile for both sexes [17], and should ideally be target-specific. A potential way to achieve this is by exploiting individual species’ dietary preferences. The nanocomposite sterilant ND-1 (Nongda-1), a compound sterilant agent whose main components are shikonin and quinestrol, had obvious effects on an experimental population of laboratory mice and midday gerbils (*Meriones meridianus*) at the concentration of 50 mg/kg [21,23]. In addition, it had antifertility effects on both males and females [21,23]. The nano-sterile agent ND-1 significantly increased the absorption of drugs in laboratory mice, therefore maintaining a significant fertility effect on females and males by reducing the concentration of drugs used [23]. However, the effect of ND-1 on the control of fertility in wild rodent populations remains unclear.

Midday gerbils (*Meriones meridianus*) are the most common rodents found in the desert steppe, desert, and agricultural areas of northern China. They are a social rodent species with strong adaptability and no night-time hibernation [24]. They have two breeding peaks in spring and autumn. After mass reproduction, the population steals and stores many plant seeds, destroys the soil structure, harms the vegetation of the habitat, and leads to continuous degradation of the desert ecosystem [25,26]. Additionally, as the main hosts of *Yersinia pestis* and *Leishmania* spp., midday gerbils can spread diseases and cause plagues, which is a key reason for rodent control.

We studied the wild population of midday gerbils in the Alxa Desert of Inner Mongolia, China, and a nanoscale sterilant ND-1 bait with a concentration of 30 mg/kg was used to conduct a field semi-closed plot fertility control test from 2018 to 2019. By monitoring the wild population structure, sex ratio, dynamic changes, and development trends of the population number and individual home range changes of midday gerbils, the effect of the nanoscale sterilant ND-1 on the growth of wild populations of midday gerbils was clarified. This was conducted with the aim of providing a practical and environmentally friendly formulation for the control of rodent fertility.

## 2. Study Site and Research Methods

### 2.1. Natural Overview of the Study Area

The research site is located in a typical desert area in southern Alashan, Inner Mongolia, China, Alxa Left Banner. The geographical coordinates are E 104°10′~105°30′, N 37°24′~38°25′, and the average altitude is 800~1500 m. The grassland type is predominantly desert and semi-desert grassland, which forms part of temperate desert arid areas. The climate is typical continental, characterized by sufficient sunshine, strong evaporation, drought, and lower rainfall [27]. The average annual precipitation is 80–220 mm, the annual evaporation is 2900–3300 mm, and the annual average temperature is 7.2 °C [28]. The chernozem and brown desert soils are light brown. The soils were relatively poor, with relatively little fertilizer having been used. Plant species richness was relatively poor, with low vegetation cover and little diversity in terms of vegetation structure. The vegetation in the study area had a high level of salt and drought resistance. It was mainly composed of hyperxeric, xerophytic, halophytic, semi-shrubs, and small shrubs. There were few perennial grasses and legumes, and vegetation cover was 1–20%. The common species were *Salsola tragus*, *Echinops sphaerocephalus*, *Cirsium arvense var. integrifolium*, *Grubovia dasyphylla*, *Cynanchum komarovii*, *Pennisetum flaccidum*, *Agropyron mongolicum*, *Psammochloa villosa*, and *Artemisia dubia*. Midday gerbils were the dominant rodent species [29]).

### 2.2. Research Methods and Data Collection

#### 2.2.1. Experimental Materials

The compound fertility agent ND-1 used in this study was a dosage form composed of shikonin and quinestrol. Shikonin is a naphthoquinone extracted from the roots of *Aspergillus euchroma*. The purities of the shikonin and quinestrol used in this study were greater than 99%. The formula of ND-1 bait per unit weight was as follows: 1 kg ND-1 bait = 950 g mouse diet (mouse diet was made by mixing corn, soybean meal, and white flour in equal proportions) + 3 g shikonin + 0.3 g quinestrol + 50 mL edible oil + 50 mL water.

In the spring of 2018, shikonin and quinestrol were dissolved in edible oil at a ratio of 10:1. The same amount of distilled water was added and emulsified to prepare a 30 mg/kg ND-1 nanoemulsion with the main component particles being below 100 nm. The ND-1 nanoemulsion was fully mixed at a concentration of 30 mg/kg with the standard mouse diet base to make a round rod diet (length 1.5–2 cm, diameter 4–5 mm).

#### 2.2.2. Sample Settings

**Figure 1:**
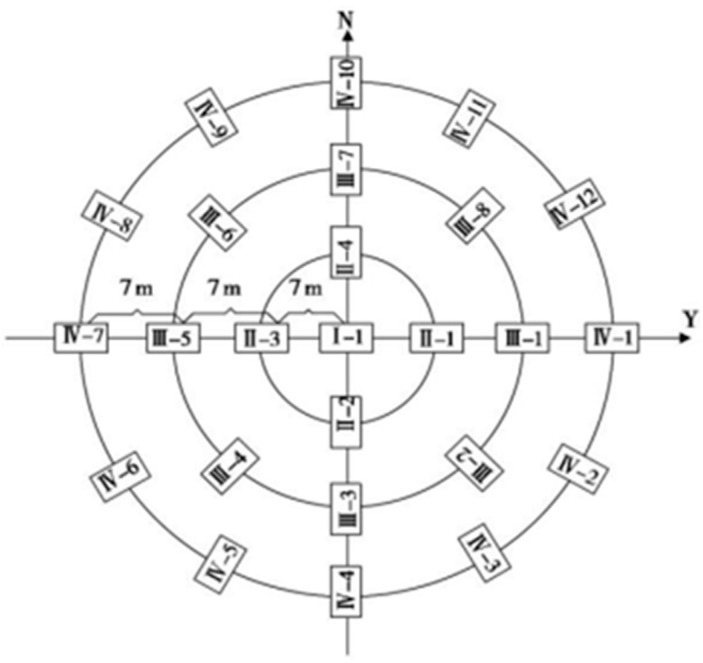
Plot location map of cages.

In April 2018, three experimental plots were selected from the same habitat type in a representative desert area of southern Alxa Left Banner as the sterile agent test areas (A, B, C) and an equal area as the control area (CK), for a total of four plots, each with a plot area of 1.5 hm^2^. A steel wire fence and an asbestos tile were used as fences to close the plots. The ground height was 90 cm and the depth underground was 60 cm to prevent rodents from escaping after digging holes. There were only midday gerbils present in the sample area, with no other rodents or animals present. An isolation buffer zone with a width of more than 100 m was set between the plots, and the isolation buffer zone was not sampled. Each plot was divided into three groups of standard quadrats for repeated sampling, with a standard quadrat area of 0.138 hm^2^. The quadrat setting followed the principle of random and uniform, and considered the relative density of midday gerbil holes, using concentric circular cages (Figure 1). A group of concentric circles was established in each standard quadrat. Each group of concentric circles was composed of three concentric circles of different sizes, and 25 cages were used. The center of the concentric circle was used to select the location of the concentrated area of the home range. There were 1, 4, 8, and 12 cages from the inside to the outside. The radius difference of each concentric circle was 7 m, and the cages were evenly distributed with east, south, west, and north as the main azimuth axes. Starting from the east azimuth axis, the cages were numbered by outward diffusion from the center of the circle.

#### 2.2.3. Assessment of Feeding Sterile Bait

On 1 April 2018, the bait feeding assessment was conducted in three plots in the sterile test area. A total of 300 g of the sterile ND-1 bait was selected and placed in a bait box. In each plot, 10 feeding points were set up (the feeding points coincide with the quadrat position of the population survey), and bait boxes (spaced at 10 m intervals) were placed according to the sample line. Each bait box was placed with 10 g sterile bait, and there was a total of 30 feeding points. The remaining sterile bait after 24 h, 48 h, 72 h, 96 h, and 120 h was detected and recorded. After weighing and measuring, 13 bait boxes had a residual amount of 0 g after 24 h, and the contents of 30 bait boxes had been consumed after 96 h, that is, the feeding rate reached 100% after 4 d. After the assessment of sterilant bait feeding in the experimental area on 6 April 2018, the artificial delivery was used at a concentration of 30 mg/kg nano-scale compound sterilant ND-1 bait at a dosage of 1200 g/hm^2^ (not all midday gerbil individuals in the area consumed sterilants, with both normal individuals and individuals affected by sterilants present). Before the sterilant ND-1 was put in, the sample sizes of each index of midday gerbil population in the different areas were the same (Table S1). After delivering sterilant for 7 d, a population survey was initiated.

#### 2.2.4. Animal Data Collection

From April to mid-October annually from 2018 to 2019, the midday gerbil population was marked and recaptured in the experimental and control areas, and consecutive surveys were conducted for 4 d each month. The first midday gerbils captured were subcutaneously injected with a PIT (passive integrated transponder) chip directly above the neck, and each PIT chip was numbered uniquely. During the sampling process, the capture date, location, number of injection chips, sex, body weight (accurate to 0.1 g), male testis decline, female abdominal characteristics, vaginal opening status, and nipple characteristics were recorded. After the recording, the captured midday gerbils were released in situ.

#### 2.2.5. Age Classification of Midday Gerbil Populations

In this study, midday gerbils were divided into three age groups according to weight, namely Stage I (juveniles), Stage II (sub-adults), and Stage III (adults). Based on the weight, reproductive status, and reproductive organ development degree of male and female midday gerbils captured in 2018–2019, combined with results from relevant researchers [30,31,32], the age group classification criteria for midday gerbils were as follows: juvenile < 35 g, 35 g ≤ sub-adult < 42 g, adult ≥ 42 g.

The reproductive characteristics of female midday gerbils were mainly analyzed during the period of vaginal development. The vaginal opening was used as a discriminant index. If the vagina was open, ovarian activity had begun, which is a sign of sexual maturity [33]. Using the decline of the testis in male midday gerbils as a criterion for determining whether it was in the reproductive stage, if the testis had already declined, indicating that the testis had completely declined into the scrotum and that mature sperm were present in the epididymis, it was a sign of sexual maturity [25]. During the breeding period for this experiment, female and male midday gerbils conformed to the above reproductive characteristics.

#### 2.2.6. Midday Gerbil Activity Distance

In this study, the maximum distance method was used to measure the home range of midday gerbils. The principle of the maximum distance method was the same as that of the minimum area method and the surrounding area method [30,34]. Compared with other home range calculation methods, the maximum distance method requires fewer recaptures, is simpler to operate, is easier to calculate, and can be used to perform significance analysis [34].

The calculation formula of the maximum distance method is:

H = L_se_ + (L_s+1_ + L_e+1_)/2 (1)

where H represents the maximum distance, L_se_ is the distance between the two longest capture points in the same capture period, L_s+1_ is the distance between the starting point and the nearest trap (for rodents), and L_e+1_ is the distance between the endpoint and the nearest trap (for rodents) [35,36].

The calculation formula of the home range is S = (H/2) 2×π.

The maximum distance calculated in this study was the distance of midday gerbil activity during the 4 d capture period per month, and the monthly data were combined by year.

### 2.3. Data Processing

Data analysis was carried out using Office 2016 and SAS 9.4 (Version 9.4, SAS Institute, Inc., Cary, NC, USA) software. When the effect values of related indices changed with years and seasons were discussed, a multi-factor mixed effect model with the index as the dependent variable, the block as the random effect, and the time, area (experimental and control) and their interactive items as fixed effects was fitted, and we constructed interaction terms for the year, season, and group. Assuming that different groups change with the season each year, the differential effect was determined by fixing the level of the two variables to view the estimate of the third variable, and the difference table of effect values of the index between the experimental area and the control area was obtained at a fixed time. Due to the imbalance between areas, we used the Scheffe method to correct the confidence interval and *p* value when calculating the between-group mean difference effect, thereby reducing the false discovery rate (FDR) of multiple comparisons [37,38]. Origin software was used for plotting. The mean data were expressed as mean ± standard error (Mean ± SE). The significance level was *p* = 0.05. In this experiment, April and May were spring, June, July, and August were summer, and September and October were autumn. The population and reproductive characteristics of midday gerbils were determined in spring, summer, and autumn from 2018 to 2019:

Field feeding rate (palatability) = feeding amount/total dosage × 100%(2)

Capture rate = number of captured midday gerbils/(number of traps × number of captured days) × 100%(3)

Female capture rate in the breeding season = number of females in the breeding season/(number of traps × number of captured days) × 100%.(4)

Capture rate of male rats in the breeding season = number of male midday gerbils in the breeding season/(number of traps × number of days) × 100%.(5)

Sex ratio = female number/male number(6)

The proportion of juvenile midday gerbils = number of captured juvenile midday gerbils/number of captured midday gerbils × 100%(7)

The proportion of sub-adult midday gerbils = number of captured sub-adult gerbils/number of captured midday gerbils × 100%(8)

The proportion of adult midday gerbils = number of captured adult midday gerbils/number of captured midday gerbils × 100%(9)

## 3. Results and Analysis

### 3.1. Effect of ND-1 on the Number of Female Midday Gerbils during the Breeding Period

**Figure 2:**
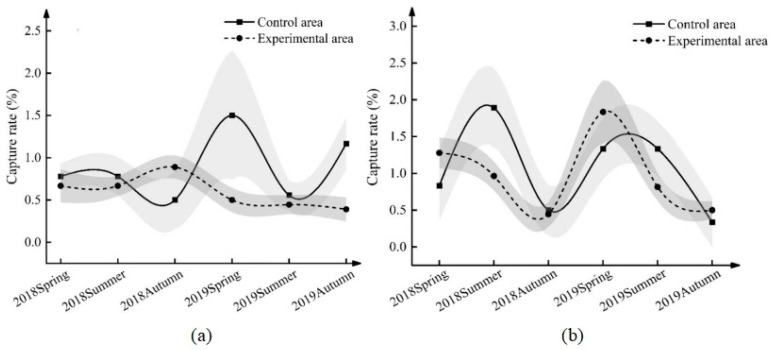
Capture rate of midday gerbils during the breeding period ((**a**): female; (**b**): male. Gray parts are banded error lines.

The number of female midday gerbils during the breeding period of the field experiment is shown in Figure 2. The results of type III analysis of the variance of the female capture rate during the breeding period in this study (Table 1 and Table S3) show that there was an interactive effect between groups and seasons and years (*F* = 3.203, *p* = 0.003). Further comparing the average value (control area vs. experimental area), the capture rate of female gerbils in the breeding period in the control area in the spring of 2019 and autumn of 2019 was significantly higher than that in the experimental area (Figure 2a, Table 2). The capture rate of female gerbils in the breeding period in the control area in the summer of 2019 was 1% (0.341~1.659%) higher than that in the experimental area; in the autumn of 2019, the capture rate of female gerbils in the breeding period in the control area was 0.778% (0.119~1.437%) higher than the experimental area. The number of female midday gerbils in the control area fluctuated considerably during the breeding period, and the capture rate curve was bimodal, with two growth peaks of 1.5% in the spring of 2019 and 1.17% in the autumn. However, the fluctuation in the number of female midday gerbils during the breeding period in the experimental area was relatively gentle, and there was only one growth peak, that is, 0.89% in the autumn of 2018. The peak number of female midday gerbils was reduced to one in the breeding period in the experimental plots, and the number of female midday gerbils in the breeding period decreased significantly.

### 3.2. Effect of ND-1 on the Number of Male Midday Gerbils during the Breeding Period

The dynamics of the number of male midday gerbils during the breeding period are shown in Table 1, Table 2 and Table S4 and in Figure 2b. In the summer of 2018, the male gerbil capture rate in the breeding period in the control area was 0.926% (0.100~1.752%) higher than that in the experimental area, and there was no significant difference between the control area and the experimental area in other periods (*p* > 0.05). However, from the dynamic number of male midday gerbils in the breeding period, there were two peaks in the control area of the capture rate curve, which were 1.89% and 1.33% during the summers of 2018 and 2019, respectively. However, there was one peak in the experimental area, that is, in the spring of 2019, when the capture rate was 1.83%. 

### 3.3. Effect of ND-1 on the Sex Ratio of Midday Gerbils during the Breeding Period

**Figure 3:**
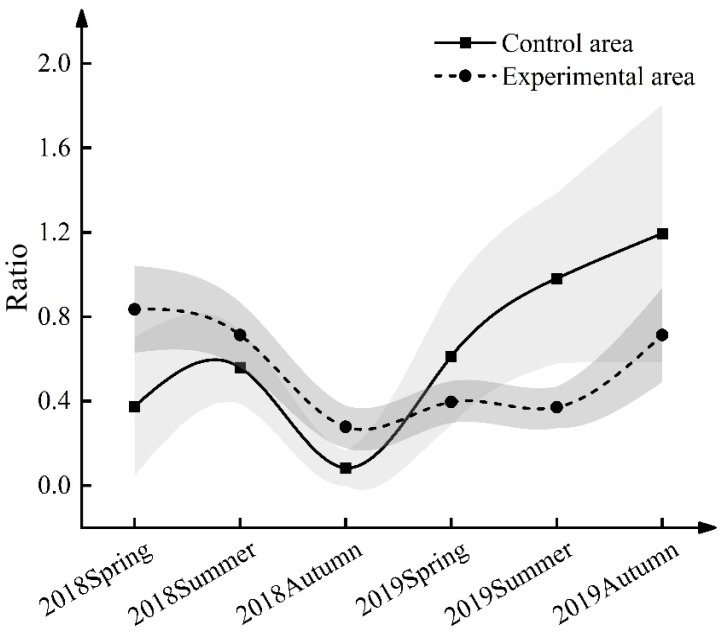
Sex ratio of midday gerbils from 2018 to 2019. Gray parts are banded error lines.

The sex ratio (♀/♂) of midday gerbils in the control area and the experimental area from 2018 to 2019 was analyzed (Table 3, Table 4 and Table S5 and Figure 3). The results of the Type III analysis of variance show that there was an interaction between groups and seasons and years (*F* = 2.581, *p* = 0.015) (Table 3). Further comparison of the average value (control area vs. experimental area) shows that there was no significant difference in the sex ratio of midday gerbils between the control and experimental areas in 2018 (Table 4, Figure 3). The seasonal sex ratio was less than 1, indicating that the midday gerbil population had a male deviation phenomenon during that year, that is, the number of male individuals was higher than that of female individuals. In the summer of 2019, the sex ratio of midday gerbils in the control area reached 0.98, which was significantly higher than that in the experimental area at the same time (*p* < 0.05) (Table 4, Figure 3). In the autumn of 2019, although there was no significant difference between the sex ratio of midday gerbils in the control area and the control area, it also reached 1.19 (approaching 1:1), while the sex ratio of midday gerbils in all seasons of the experimental area did not reach 1 in 2019. The contribution of females is generally higher than that of males during population growth. The increase in the number of females in the control area in 2019 indicates an increase in the reproductive potential of the population. The ratio of females to males decreased in the experimental area in the summer of 2019, limiting their reproductive potential.

### 3.4. Effect of ND-1 on the Age Structure of Midday Gerbil Populations

**Figure 4:**
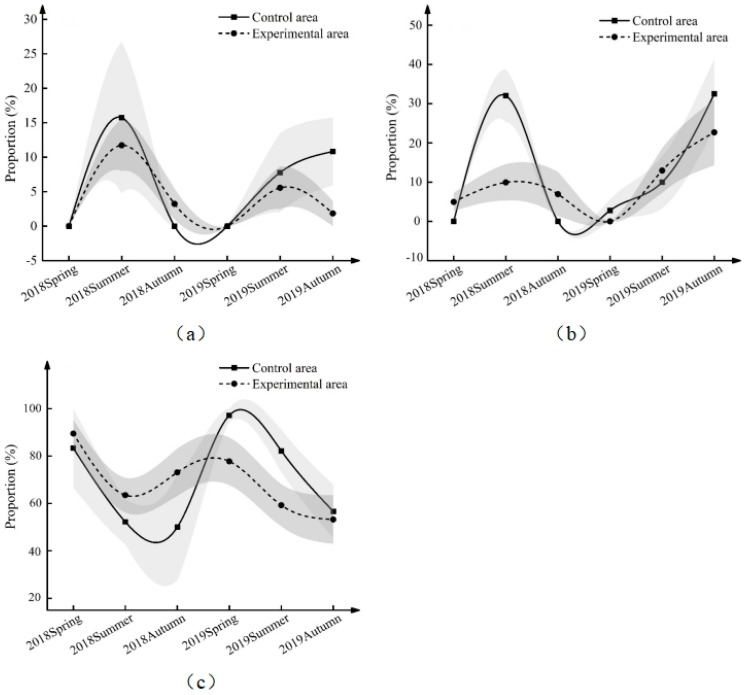
Proportion of different age structures of midday gerbils from 2018 to 2019 ((**a**): juvenile; (**b**): sub-adult; (**c**): adult. Gray parts are banded error lines.

We analyzed the changes in the proportion of each age group in the control and experimental areas, as shown in Figure 4 and Table 5 and Table 6. The results of type III analysis of variance for the proportion of juvenile midday gerbils (Table 5 and Table S6) show that there was no interaction effect between the group and the season and the year (*F* = 0.721, *p* = 0.654). Furthermore, the average value was further compared (control area vs. experimental area), and there was no significant difference between the control area and the experimental area in each season (Table 6, Figure 4a). However, from the summer of 2019 to the autumn of 2019, the proportion of juveniles increased in the control area but decreased in the experimental area.

The results of type III analysis of variance for the proportion of sub-adult midday gerbils (Table 5 and Table S7) show that there was an interactive effect between the group and the season and the year (*F* = 2.354, *p* < 0.05). Further comparison of the average value (control area vs. experimental area) (Table 6, Figure 4b) showed that the sub-adult ratio in the control area was 22.09% (5.172~39.025%) higher than that in the experimental area in the summer of 2018, and there was no significant difference in the sub-adult ratio between the control area and the experimental area in the rest of the time (*p* > 0.05). In 2019, the proportion of subadults in the control and experimental areas showed an upward trend. However, the increase was greater in the control area. 

The results of type III analysis of the variance of the adult ratio of midday gerbils show (Table 5 and Table S8) that there was no interaction between groups and seasons and years (*F* = 1.031, *p* = 0.412). Furthermore, the average value was further compared (control area vs. experimental area), and there was no significant difference in the adult ratio between the control area and the experimental area in each season (Table 6, Figure 4a). In summary, the age structure of the wild population of midday gerbils changed in the experimental area, such that the proportion of sub-adults in the current year, that is, the proportion of breeding subjects in the next year, was significantly lower than that in the control area. This inhibited the breeding base for future population growth.

### 3.5. Effects of ND-1 on Midday Gerbil Populations

**Figure 5:**
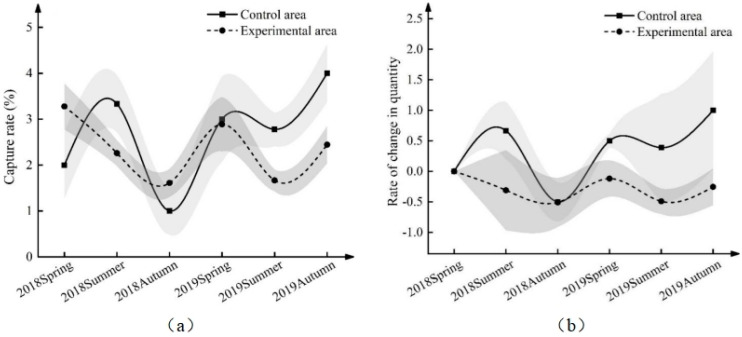
Population and growth rate of midday gerbils from 2018 to 2019 ((**a**): population; (**b**): growth rate). Gray parts are banded error lines.

The population dynamics of midday gerbils during the two years of the field experiment are shown in Table 7, Table 8, Tables S9 and S10 and in Figure 5. The results of type III variance analysis of the population number and population growth rate of midday gerbils show that there were interactive effects between groups and seasons and years (*F* = 2.827, *p* < 0.05; *F* = 5.104, *p* < 0.05). Further comparison of the average value (control area vs. experimental area) shows that the population of midday gerbils in the control area was significantly higher than that in the experimental area in the autumn of 2019 (*p* < 0.05), but there was no significant difference between the control area and the experimental area in other periods (Table 8, Figure 5a). The results of the population growth rate of midday gerbils show that the control area was higher than the experimental area in the summer of 2018 and in the spring, summer, and autumn of 2019, and the difference is statistically significant (*p* < 0.001). Among them, the control area in the summer of 2018 was 0.997% (0.560~1.395%) higher than the experimental area. Compared with the experimental area, the control area was 0.619% (0.107~1.130%) higher in spring, 0.880% (0.463~1.298%) higher in summer and 1.254% (0.743~1.766%) higher in autumn in 2019 (Table 8, Figure 5b). The growth rate in the control area fluctuated greatly. The growth rate of midday gerbils in the experimental area fluctuated slightly and the curve was relatively flat. In the range of −0.51% to −0.12%, the growth rate was negative.

### 3.6. Effects of ND-1 on Nesting Areas of Midday Gerbil Populations

Home range was calculated after statistical analysis of the habitat activity radius of midday gerbils during the two-year study period (Table 9). It can be seen that as far as female midday gerbils are concerned, the average nest area of the control area was the largest in 2018, reaching 2509.43 m^2^, the nest area of female midday gerbils in the experimental area in the same year was 1750.35 m^2^, and there was no significant difference between them (*F* = 0.701, *p* > 0.05). At the same time, there was no significant difference between the control area and the experimental area in 2019 (*F* = 1.725, *p* > 0.05). There was also no significant difference in the nest area of male midday gerbils between the two years (*p* > 0.05). 

## 4. Discussion

Population age structure is a characteristic of animal populations. Different age structures can reflect the reproductive growth of the population in the current year and the next year to a certain extent and indirectly determine the development trend of the population [39]. The change in age composition and its range play a decisive role in determining the potential and size of the population. Midday gerbils have a short ecological life, a long breeding period, and more young midday gerbils occur in summer and autumn. Midday gerbils have the characteristics of high fecundity in summer and autumn to compensate for the high mortality in winter. When the environmental conditions in winter are relatively deteriorated, juvenile midday gerbils will be eliminated naturally, and the number will decrease [40]. 

Our previous studies [21,23] showed that the non-nanosterile agent ND-1 delays the breeding start-up period of midday gerbils and lab mice under laboratory conditions, prolongs their breeding cycle, reduces the annual breeding times and breeding rate, and delays the breeding start-up period [21,23]. It significantly interferes with the population structure, affects the overwintering survival rate and the next year’s breeding base of the offspring, and is conducive to the sustainable control of the population. In the present study, ND-1 effectively interfered with the age structure of a wild population of midday gerbils in the experimental area. The age structure composition changed significantly, the peak of population reproduction moved backward, and the number of sub-adult midday gerbils born during the summer high-reproduction period decreased. Sub-adult midday gerbils born in summer and autumn are the main bodies of spring reproduction in the following year. The survival rate of the sub-adult population in autumn or winter is much lower than that of adults [41], which in turn affects the fitness between individuals of the midday gerbil population.

Changes in the sex ratio affect the population structure, community composition, and mating relationships of animals, as well as mating competition, reproductive investment, and reproductive success [42,43]. During the breeding process, the sex ratio indirectly determines the developmental trends of the population [42]. Studies have shown that Bayo sterilant changed the sex ratio of the Brandt’s vole (*Lasiopodomys brandtii*) population, making the sex ratio of Brandt ‘s voles more stable and less volatile, such that the population of Brandt’s voles remained at a low level [44]. The mating system of the midday gerbil population is a mixed system based on monogamy [45,46], and the distribution of pairs and the availability of resources affect the outcome of the mating system. Imbalances in effective sex ratios, such as a shortage of females, can lead to a monogamous mating strategy where males cannot mate with multiple females [45].

**Figure 6:**
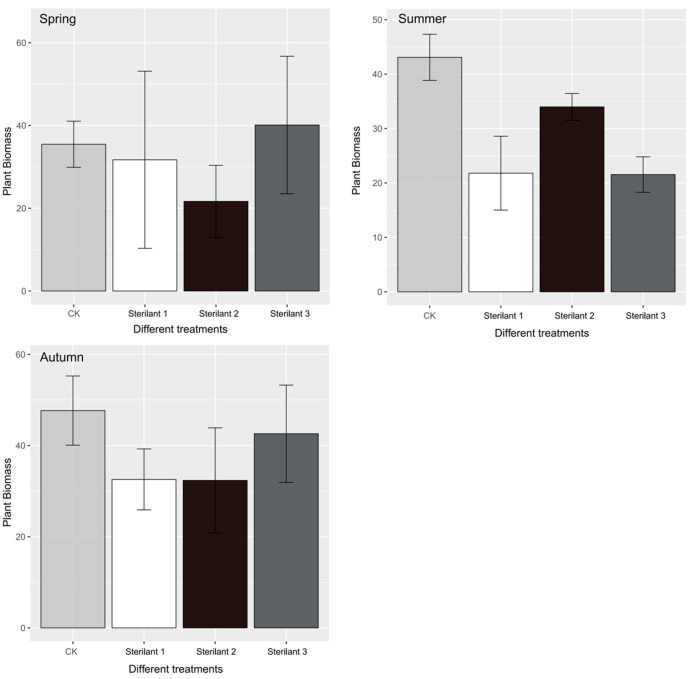
Comparison of plant biomass in different areas.

In 1930, Fisher pointed out that since every individual from sexual reproduction has only one father and one mother, the reproductive value of the male parent and the female parent is equal in general. Therefore, if the sex ratio of the population deviates from the same number of females and males, the scarce sexes will have a selective advantage until the sex ratio is equal [47]. For dioecious animals, environmental factors and time factors have certain regulatory effects on the population sex ratio [48]. In 2018, the sex ratios of midday gerbils in the control area and the experimental area were less than 1, namely the number of female midday gerbils was less than that of male midday gerbils. After internal adjustment for 1 year, the sex ratio of midday gerbils in the control area approached 1:1 in 2019, which was consistent with Fisher’s sex ratio theory [47], and the sex ratio of midday gerbils in each season tended to be stable, while the sex ratio of midday gerbils in the experimental area did not reach 1 after one year, and the sex ratio of midday gerbils in the experimental area was significantly lower than that in the control area in the summer of 2019. In this study, the environmental factors such as soil and climate were very similar between the experimental area and the control area. At the same time, we conducted one-way ANOVA on the biomass of herbaceous plants in different blocks (Figure 6), and the results show that there was no significant difference in herbaceous biomass among different regions (spring: *F* = 0.293, *p* = 0.83; summer: *F* = 3.538, *p* = 0.0679; autumn: *F* = 0.662, *p* = 0.598). This indicates that the availability of food resources was consistent across the four blocks. This will highlight that a reduction in the number of females was not a consequence of food availability. 

The change in sex ratio also corresponded to the change in the number of female midday gerbils in the breeding period of each season. In 2018, there was no significant difference between the number of female midday gerbils in the breeding period of the control area and the experimental area, and the fluctuation of the number in each season was relatively gentle. Until the spring and autumn of 2019, the number of female midday gerbils in the breeding period in the control area increased rapidly and was significantly higher than that in the experimental area in the same period. The effect of the sterilant on female midday gerbils in the reproductive period appeared as hysteresis, which may be due to the fact that the sterilant was put into use in April, and the mating behavior of midday gerbils starts in early March [49]. The sterilant did not affect the reproduction of female midday gerbils in the spring of that year, but the sterilant ND-1 would prolong the reproductive cycle of female midday gerbils [21], that is, reduce the reproductive frequencies of female midday gerbils. Midday gerbils in the experimental area could not adjust the population sex ratio through multiple reproduction, which is the reason why the sex ratio in the experimental area was still unbalanced in 2019. 

The inhibition of the sex ratio in the experimental area means that the number of female midday gerbils in the population remained at a low level, and the population growth potential was inhibited. In the process of population growth, the contribution of females to population growth is greater than that of males [50]. The sterilant ND-1 reduced the number of females during the breeding period in the experimental area, which caused the midday gerbil population to decline, thus maintaining a low level.

The home range is an area where animals often move to meet their daily needs such as feeding, reproduction, and young rearing [51]. Home range changes reflect life-history strategies and population dynamics [41]. Currently, few studies have been conducted on the effects of fertility agents on rodent nests using field experiments. The nano-compound sterilant ND-1 used in the present study had no significant effect on the home ranges of male and female midday gerbils. The results show that, although the sterilant reduced the reproductive capacity of male and female midday gerbils, it had no significant effect on their activity range or ability. In other words, infertile individuals can completely rely on their own ability to compete with fertile individuals for mates, which leads to a decrease in the overall reproduction rate of the population and a continuous decrease in the population size [52]. On the other hand, infertile individuals can still occupy the same home range and field as fertile individuals in the year of drug administration by their own activity ability, which will not weaken the internal competition of the population in space, so that the density of the unit space of the midday gerbil population with density-dependent population regulation mechanisms will not decrease, and competition will continue, which is also a major factor in the continuous decrease of the population in the next year.

Therefore, the one-time release of nano-scale compound sterilant ND-1 in spring can alter the reproductive patterns of wild populations of midday gerbils. Compared to natural populations, there is a noticeable shift in the peak of reproduction, and significant changes occur in population structure and population dynamics. This clearly demonstrates that the nano-scale compound sterilant ND-1 has a significant and sustained control effect on the population growth of midday gerbils.

## Figures and Tables

**Table 1:** Fixed-effects III-type test—the capture rate of midday gerbils during the breeding period.

Sex	Effect	F	*p*
Female	Group	1.504	0.222
	Season	0.338	0.714
	Year	1.878	0.173
	Group × Season × Year	3.203	0.003
Male	Group	0.108	0.743
	Season	7.615	0.001
	Year	0.041	0.839
	Group × Season × Year	1.637	0.129

**Table 2:** Differential effect of capture rate of midday gerbils (males and females were modeled separately).

Effect	Sex	Season	Year	Estimate (%)	SE (%)	*t*	*p*	95% CI
Control vs. Experimental	Female	Spring	2018	0.111	0.144	0.459	0.613	0.479~1.444
			2019	1.000	0.334	2.998	0.003	0.341~1.659
		Summer	2018	0.111	0.272	0.408	0.684	−0.427~0.649
			2019	0.111	0.272	0.408	0.684	−0.427~0.649
		Autumn	2018	−0.389	0.334	−1.166	0.245	−1.048~0.270
			2019	0.778	0.334	2.332	0.021	0.119~1.437
	Male	Spring	2018	−0.444	0.512	−0.868	0.387	−1.456~0.567
			2019	−0.500	0.512	−0.977	0.330	−1.511~0.511
		Summer	2018	0.926	0.418	2.215	0.028	0.100~1.752
			2019	0.519	0.418	1.240	0.217	−0.307~1.344
		Autumn	2018	0.056	0.512	0.109	0.914	−0.956~1.067
			2019	−0.167	0.512	−0.326	0.745	−1.178~0.845

**Table 3:** Fixed-effects III-type test—sex ratio of midday gerbils.

Effect	F	*p*
Group	0.368	0.545
Season	0.248	0.781
Year	2.979	0.086
Group × Season × Year	2.581	0.015

**Table 4:** Differential effect of sex ratio of midday gerbils.

Effect	Season	Year	Estimate	SE	*t*	*p*	95% CI
Control vs. Experimental	Spring	2018	−0.460	0.357	−1.290	0.199	−1.165~0.245
		2019	0.216	0.357	0.605	0.546	−0.489~0.921
	Summer	2018	−0.154	0.291	−0.528	0.599	−0.729~0.422
		2019	0.611	0.291	2.098	0.038	0.036~1.187
	Autumn	2018	−0.194	0.357	−0.545	0.587	−0.899~0.510
		2019	0.481	0.357	1.349	0.179	−0.223~1.186

**Table 5:** Fixed-effects III-type test—proportion of different age structures of midday gerbils.

Age Structure	Effect	F	*p*
Juvenile	Group	0.615	0.434
	Season	6.008	0.003
	Year	0.094	0.759
	Group × Season × Year	0.721	0.654
Sub-adult	Group	0.669	0.415
	Season	5.110	0.007
	Year	1.245	0.266
	Group × Season × Year	2.354	0.026
Adult	Group	0.015	0.902
	Season	5.580	0.005
	Year	0.120	0.730
	Group × Season × Year	1.031	0.412

**Table 6:** Differential effect of the proportion of different age structures of midday gerbils.

Effect	Age Group	Season	Year	Estimate (%)	SE (%)	*t*	*p*	95% CI
Control vs. Experimental	Juvenile	Spring	2018	−0.000	6.613	−0.000	1.000	−13.065~13.065
			2019	−0.000	6.613	−0.000	1.000	−13.065~13.065
		Summer	2018	4.012	5.400	0.743	0.459	−6.655~14.680
			2019	2.222	5.400	0.412	0.681	−8.445~12.890
		Autumn	2018	−3.241	6.613	−0.490	0.625	−16.306~9.824
			2019	8.981	6.613	1.358	0.176	−4.083~22.046
	Sub-adult	Spring	2018	−4.960	10.494	−0.473	0.637	−25.691~15.770
			2019	2.778	10.494	0.265	0.792	−17.953~23.508
		Summer	2018	22.099	8.568	2.579	0.011	5.172~39.025
			2019	−2.963	8.568	−0.346	0.730	−19.889~13.963
		Autumn	2018	−6.944	10.494	−0.662	0.509	−27.675~13.786
			2019	9.815	10.494	0.935	0.351	−10.916~30.545
	Adult	Spring	2018	−6.151	18.368	−0.335	0.738	−42.436~30.134
			2019	19.444	18.368	1.059	0.291	−16.841~55.729
		Summer	2018	−11.296	14.997	−0.753	0.452	−40.923~18.330
			2019	22.963	14.997	1.531	0.128	−6.664~52.590
		Autumn	2018	−23.148	18.368	−1.260	0.209	−59.433~13.137
			2019	3.426	18.368	0.187	0.852	−32.859~39.711

**Table 7:** Fixed-effects III-type test of population and growth rate of midday gerbils.

	Effect	F	*p*
Population	Group	1.105	0.295
	Season	0.854	0.428
	Year	3.117	0.079
	Group × Season × Year	2.827	0.008
Growth rate	Group	39.067	<0.001
	Season	0.893	0.412
	Year	7.858	0.006
	Group × Season × Year	5.104	<0.001

**Table 8:** Differential effect of population and growth rate of midday gerbils.

Effect		Season	Year	Estimate (%)	SE (%)	*t*	*p*	95% CI
Control vs. Experimental	Population	Spring	2018	−1.278	0.808	−1.580	0.116	−2.875~0.319
			2019	0.111	0.808	0.137	0.891	−1.486~1.708
		Summer	2018	1.074	0.660	1.627	0.106	−0.230~2.378
			2019	1.111	0.660	1.683	0.094	−0.193~2.415
		Autumn	2018	−0.611	0.808	−0.756	0.451	−2.208~0.986
			2019	1.556	0.808	2.124	0.037	−0.042~3.153
	Growth Rate	Spring	2018	<0.001	0.259	<0.001	1.000	−0.512~0.512
			2019	0.619	0.259	2.388	0.018	0.107~1.130
		Summer	2018	0.977	0.212	4.621	<0.001	0.560~1.395
			2019	0.880	0.212	4.163	<0.001	0.463~1.298
		Autumn	2018	0.008	0.259	0.033	0.974	−0.503~0.520
			2019	1.254	0.259	4.842	<0.001	0.743~1.766

**Table 9:** The area of midday gerbil home range from 2018 to 2019 (unit: m^2^).

Year	Female				Male			
	Control	Experimental	*F*	*p*	Control	Experimental	*F*	*p*
2018	2509.43 ± 380.08	1750.35 ± 175.20	0.701	0.058	2681.55 ± 445.92	1919.79 ± 297.65	1.945	0.174
2019	1424.92 ± 380.10	2079.63 ± 322.40	1.725	0.197	1820.16 ± 340.55	2276.46 ± 286.11	0.937	0.339

## Data Availability

The data presented in this study are available in supplementary materials here.

## Supplementary Materials

The following supporting information can be downloaded at https://www.mdpi.com/article/10.3390/life13122280/s1. Table S1: Sample sizes for different indicators of the midday gerbil population in April 2018; Table S2: Sample sizes of different indicators of midday gerbil populations in different seasons; Table S3: Fixed effect of female midday gerbils during the breeding period; Table S4: Fixed effect of male midday gerbils during the breeding period; Table S5: Fixed effect of the proportion of sex ratio of midday gerbils; Table S6: Fixed effect of the proportion of juvenile midday gerbils; Table S7: Fixed effect of the proportion of sub-adult midday gerbils; Table S8: Fixed effect of the proportion of adult midday gerbils; Table S9: Fixed effect of population of midday gerbils; Table S10: Fixed effect of growth rate of midday gerbils.
